# Non-Invasive Photodynamic Therapy against -Periodontitis-causing Bacteria

**DOI:** 10.1038/s41598-019-44498-4

**Published:** 2019-06-03

**Authors:** Danbi Park, Eun Joo Choi, Kwon-Yeon Weon, Wan Lee, Seoung Hoon Lee, Joon-Seok Choi, Gyu Hwan Park, Bada Lee, Mi Ran Byun, Kyunghwa Baek, Jin Woo Choi

**Affiliations:** 1 Department of Pharmacology, College of Dentistry and Research Institute of Oral Science, Gangneung-Wonju National University, Gangwon-do, 25457 Republic of Korea; 2 School of Dentistry and Dental Research Institute, Wonkwang University, Iksan, Choenbuk 54538 Republic of Korea; 3 College of Pharmacy, Daegu Catholic University, Gyeongbuk, 38430 Korea; 4 Research Institute of Pharmaceutical Sciences, College of Pharmacy, Kyungpook National University, Daegu, 41566 Republic of Korea; 5 Department of Pharmacology, College of Pharmacy, Kyung Hee University, Seoul, 02453 Republic of Korea; 6 Department of Life and Nanopharmaceutical Sciences, Kyung Hee University, Seoul, 02453 Republic of Korea

**Keywords:** Dental biofilms, Periodontitis

## Abstract

Periodontitis is initiated by causative bacteria in the gingival sulcus. However, as the lesion is often deep and out of circulation system and biofilm is frequently formed on the bacteria cluster, use of antibacterial agents has been limited and the invasive method such as curettage is thought as an only treatment. Here we designed non-invasive photodynamic therapy (PDT), with the ointment which leads a photosensitizer deliverable into gingival sulcus. We assessed whether 650 nm light-emitting-diode (LED) penetrates the 3-mm soft tissue and effectively activates a photosensitizer toluidine-blue-O (TBO) through the thickness to remove *Porphyromonas gingivalis* and *Fusobacterium nucleatum* species. The oral ointment formulation was optimized to efficiently deliver the photosensitizer into gingival sulcus and its efficacy of PDT was evaluated in *in vitro* and *in vivo* models. Four weeks of TBO-formulation mediated-PDT treatment significantly attenuated periodontitis-induced alveolar bone loss and inflammatory cytokines production in rats. These results confirm that a 650 nm LED indeed penetrates the gingiva and activates our TBO formulation which is sufficiently delivered to, and retained within, the gingival sulcus; thus, it effectively kills the bacteria that reside around the gingival sulcus. Collectively, TBO-mediated PDT using LED irradiation has potential as a safe adjunctive procedure for periodontitis treatment.

## Introduction

Periodontitis is a complex inflammatory disease characterized by progressive destruction of the tooth’s supporting tissues as a result of interactions between bacterial products, cell populations, and mediators. Even though the definitive mechanism remains unclear, a crucial factor in inflammation is the outgrowth of multiple opportunistic microorganisms in the oral cavity^1^. These microorganisms produce endotoxins, induce various inflammatory cytokines, and finally form dental plaque biofilms, an aggregate of microorganisms in which cells that are embedded within a self-produced matrix of extracellular polymeric substance adhere to each other or to a surface^2,3^.

Among the conventional treatments for periodontal disease, the most widely used approach is anti-infective non-surgical treatment involving the use of antibiotics aimed at controlling microorganisms, biofilm, and other prominent risk factors. However, the prolonged, long term use of systemic antibiotics exposes patients to the risk of developing antibiotics-resistant strains and superimposed infections^4,5^. For patients with advanced refractory disease, antibiotics are used in conjunction with a variety of periodontal surgical interventions to reduce the depth of periodontal pockets. Surgical treatment of periodontitis has been fairly successful, however, is an invasive procedure with a high recurrence rate in patients. In addition, the delivery of antibiotics to the lesions with appropriate concentration is difficult because of anatomical characteristics of surrounding tissues and the biofilms formed by microorganisms and mediators^6^.

The shortcomings of conventional treatments for periodontal disease have led to the proposal of adjunctive antimicrobial photodynamic therapy as an alternative treatment. When a nontoxic photosensitizing chemical substance is activated by light of a specific wavelength, it produces reactive oxygen species (ROS) that can damage DNA and cell membranes to elicit a microbial killing effect^7,8^. Moreover, PDT appears to obstruct biofilm differently from general antibiotics^9,10^.

Toluidine blue O (TBO) is a cationic phenothiazine dye that has been comprehensively investigated as an antibacterial photosensitizer, and it interacts with lipopolysaccharide (LPS), the major component of the gram-negative bacterial outer membrane. Periodontal pathogenic bacteria are known to be susceptible to PDT using a light-emitting diode (LED) in the presence of TBO^11,12^. LED as a light source has the advantage of limited heat production over high-power lasers and, therefore, reduces off target thermal effects^13^. Previous studies demonstrated the bactericidal effect of TBO delivered using blue LED on periodontal pathogens including *Porphyromonas gingivalis*, *Aggregatibacter actinomycetemcomitans* and *Porphyromonas intermedia*, suggesting that the potential of TBO as a treatment for periodontal disease^11,14–16^. The light dose required to kill TBO-treated bacterial cells is much lower than that causing toxicity in cultured human keratinocytes and fibroblasts^17^.

The transmission of light is generally dependent on the thickness of the tissue sample. Light at wavelengths (WV) greater than 600–650 nm is able to penetrate a thickness of 2–3 mm tissue structure^18,19^. Given that the thickness of human gingival tissue is approximately 2–3 mm, in this study, an antibacterial photosensitizer against periodontitis-causing pathogens is designed with a wavelength between 600–650 nm. As the gingival sulcus is classified as a difficult site for drug delivery because of the exudation of effusion, which has been a challenging factor in the development of dental drugs^20^, we optimized the efficient release of the photosensitizer with ointment formulation. This may suggest a clinically beneficial prototype for periodontitis treatment.

## Results

### Antimicrobial effects of PDT penetrating tissue depth against *P. gingivalis* and *F. nucleatum*

**Figure 1:**
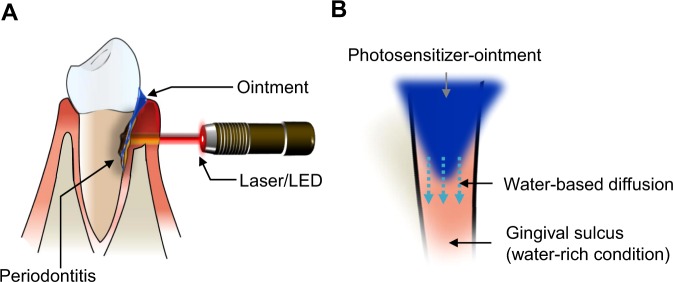
Design of PDT for periodontitis treatment. (**A**) Depicted description for PDT strategy for periodontitis treatment using photosensitizer toluidine blue O (TBO) ointment formulation. (**B**) Design of ointment for TBO to be diffused (or released) into the water rich sulcus area.

**Figure 2:**
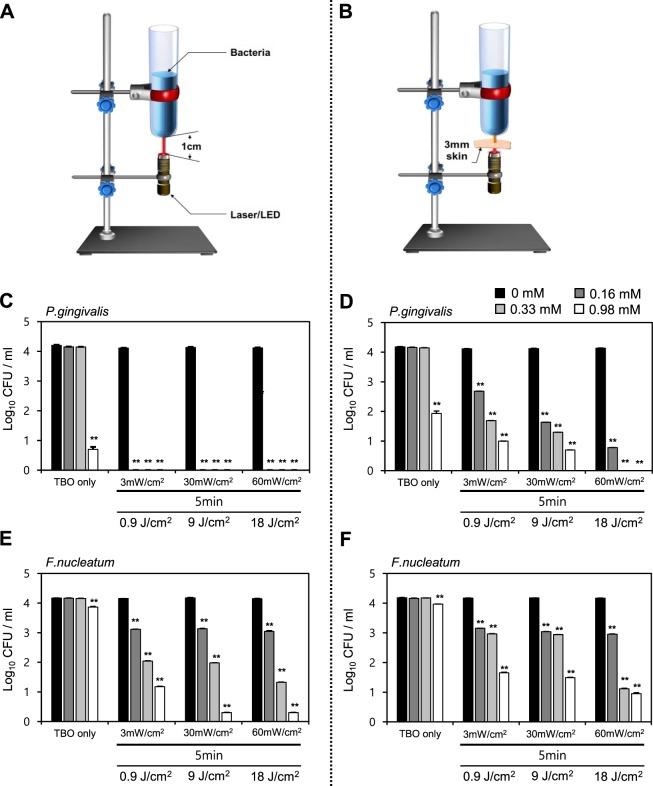
TBO with LED penetrating through soft tissue effectively kills *P*. *gingivalis* and *F*. *nucleatum* strains *in vitro*. (**A**,**B**) Design of antimicrobial test of PDT penetrating tissue depth. The 3 mm-thick artificial skin is placed between the LED device and the bacterial suspension to test whether LED irradiation at a wavelength of 650 nm penetrates through soft tissue, thereby activating the bactericidal effects of TBO. TBO in powder form was dissolved in PBS. TBO solutions were added to the bacterial suspension to obtain final concentrations of 0.16, 0.33, and 0.98 mM. After 5 min of incubation, TBO- containing wells were exposed to 3, 30, and 60 mW/cm^2^ of LED irradiation at a 650 nm wavelength for 5 min. TBO-containing bacterial suspension wells were directly exposed to LED irradiation (**C**) for *P*. *gingivalis* and (**E**) for *F*. *nucleatum*, respectively. (**D**,**F**) TBO-containing bacterial suspension wells were exposed to LED irradiation through 3 mm-thick artificial skin (**D**) for *P*. *gingivalis* and (**F**) *F*. *nucleatum*, respectively. The data represent the mean ± the standard deviation (n = 2), *p < 0.05, **p < 0.01 vs. control (without TBO and without LED exposure). CFU, viable count colony forming unit.

The penetrability of LED light at a wavelength of 650 nm through 3 mm-thick skin tissue and its activation of the antibacterial and anti-biofilm effect of TBO, may be logically considered as a critical aspect in the development of PDT for the treatment of periodontitis. Development of a topical formulation that can be applied to the oral cavity to release photosensitizer, i.e., TBO for an adequate period to allow its delivery to the target site is also necessary for PDT as an alternative to overcome the limitations of conventional treatments (Fig. 1A,B). Thus, we assessed whether LED light at a wavelength of 650 nm penetrates through the soft tissue to activate the bactericidal and anti-biofilm effects of TBO against *P*. *gingivalis* and *F*. *nucleatum* species (Fig. 2A,B). First, the bactericidal efficacy of TBO with direct LED irradiation (650 nm wavelength) was tested and the combinations of light energy and photosensitizer concentration were optimized for antibacterial PDT for periodontitis. When *P*. *gingivalis* was incubated with TBO in the dark, no influence on bacterial growth was noted at a concentration of 0.16 mM or 0.33 mM (Fig. 2C). With higher concentrations of TBO (0.98 mM), bactericidal rate was almost 99% after 48 h of incubation without light exposure. Irradiation of the supernatants in the presence of TBO achieved a high bactericidal rate (approximately 99.9%) at all tested TBO concentrations (0.16 mM, 0.33 mM, 0.98 mM) regardless of the tested light intensity or irradiation time of 1 and 5 min (Figs 2C and S1A). Irradiation with an LED had no significant effect on the viability of colony counts for *P*. *gingivalis* in the absence of TBO.

To evaluate the therapeutic potential of LED light at a wavelength of 650 nm on periodontal lesions, we assessed whether a 650 nm wavelength LED penetrates 3 mm thick artificial human skin and thereby activates the bactericidal effects of TBO. Although the bactericidal effect was reduced compared with that observed using direct irradiation, irradiation of the supernatants through 3 mm-thick artificial skin in the presence of TBO resulted in a substantial light-dose-dependent reduction in viable colony counts (Figs 2B,D and S1B).

In test with *F*. *nucleatum* incubated with TBO, no influence on bacterial growth was noted at concentrations of 0.16 mM or 0.33 mM in the absence of light (Fig. 2E). At higher concentrations of TBO (0.98 mM), bacterial growth was inhibited (−51% vs. control [CON; without TBO and without LED exposure], p < 0.05) after 48 h of incubation without light exposure. Irradiation with an LED for up to 5 min had no significant effect on the viability of colony counts for *F*. *nucleatum* in the absence of TBO. Irradiation of the supernatants in the presence of TBO resulted in a substantial light-dose and TBO-concentration dependent reduction in viable colony counts. A significant decrease in viable colony counts was observed as the TBO concentration, irradiation time, and light intensity increased. When *F*. *nucleatum* was irradiated with a 60 mW/cm^2^ LED for 5 min in the presence of 0.33 mM TBO, the bactericidal effect increased to approximately 99.9% (Figs 2E and S1C).

When the LED irradiation was performed through 3 mm-thick artificial skin, a combination of 0.33 mM TBO and LED irradiation at 60 mW/cm^2^ for 5 min revealed a decrease in viable colony counts (−99.9% vs. CON, p < 0.05) (Figs 2F and S1D). These results show that despite the slightly reduced bactericidal effect compared to the direct irradiation, a sufficient intensity of light at a wavelength of 650 nm penetrated the artificial skin to activate photosensitization.

### Biofilm-destructive effects by TBO-based PDT

**Figure 3:**
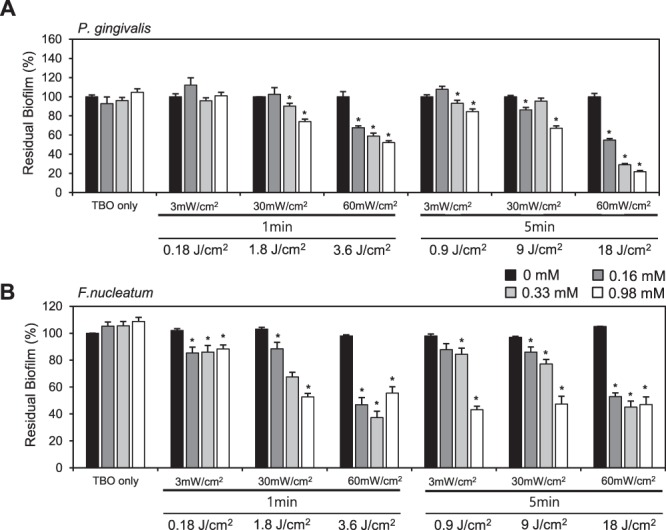
TBO with LED penetrating through soft tissue destroyed biofilms formed by *P*. *gingivalis* and *F*. *nucleatum*. The biofilm disruption by TBO with LED penetrating soft tissues. *P*. *gingivalis* (**A**) and *F*. *nucleatum* (**B**) were incubated in 96-well microtiter plates for 72 h to form a rigid biofilm. Then, TBO dissolved in PBS was added to the wells to obtain final concentrations of 0.16, 0.33, and 0.98 mM. After 5 min of incubation, TBO-containing wells were exposed to 3, 30, and 60 mW/cm^2^ of LED irradiation through 3-mm-thick artificial skin for 1 min and 5 min, followed by incubation for 24 h. The residual biofilms were quantified by crystal-violet staining. The data represent mean ± standard deviation (n = 2); *p < 0.05, **p < 0.01 vs. control (without TBO and without LED exposure).

When the LED irradiation was performed through the 3 mm-thick artificial skin, a combination of TBO and LED irradiation effectively destroyed the established 3-day old biofilms formed by *P*. *gingivalis* and *F*. *nucleatum*. In the case of *P*. *gingivalis*, when the LED irradiation was performed through the 3 mm-thick artificial skin, even a combination of 0.33 mM TBO and LED irradiation at 30 mW/cm^2^ for 1 min provided a biofilm-disrupting effect (−10% vs. CON, p < 0.05) (Fig. 3A left part). At a combination of 0.33 mM TBO and LED irradiation at 60 mW/cm^2^ for 5 min, biofilms were eliminated by almost 70% compared to the non-PDT treated control (p < 0.05) (Fig. 3A right part). In the case of *F*. *nucleatum*, when the LED irradiation was performed through the 3 mm-thick artificial skin, biofilm disrupting efficacy started to appear in the combination 0.16 mM TBO and LED irradiation at 3 mW/cm^2^ for 1 min (−15% vs CON, p < 0.05) (Fig. 3B left part). 0.33 mM TBO eliminated biofilms only light-dose-dependently, regardless of light-exposure time. A combination of 0.33 mM TBO and LED irradiation at 60 mW/cm^2^ for 1 min and 5 min caused about 63% and 55% biofilm destruction, respectively, compared to non-PDT treated control (p < 0.05) (Fig. 3B right part).

Our results showed that when the LED irradiation was performed through the 3 mm-thick artificial skin, a minimum light intensity of 3 mW/cm^2^ could provide effective photodynamic killing of *P*. *gingivalis* in the presence of 0.33 mM of TBO. For *F*. *nucleatum*, a minimum light intensity of 60 mW/cm^2^ could provide an effective bactericidal effect in the presence of 0.33 mM of TBO. This combination also showed a significant disrupting effect against the biofilm formed by *P*. *gingivalis* (−42% vs CON, p < 0.05) and *F*. *nucleatum*, respectively (−63% vs CON, p < 0.05). Considering safety issues, the most effective combination seems to be 0.33 mM of TBO and 60 mW/cm^2^ of LED irradiation with 5 min of exposure.

### Formulation retention/viscosity test and *in vitro* release study

**Figure 4:**
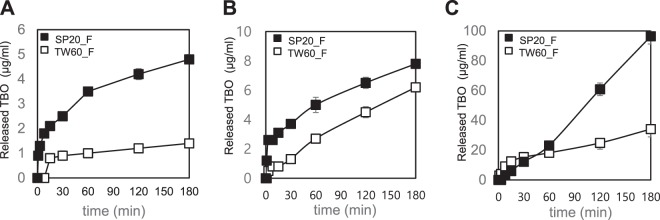
Release of TBO from SP20_F and TW60_F formulations. (**A**) TBO formulations were applied on an acrylic plate and eluted in saline medium. (**B**) TBO formulations were applied on an acrylic plate and eluted in mixed medium (100 mL of phosphate-buffered saline + 50 ml of MeOH). (**C**) TBO formulations were applied on a sponge and eluted in mixed medium (100 mL of phosphate-buffered saline + 50 ml of MeOH). SP20_F: Ointment formulation containing 75 mM of the TBO that is incorporated with Span20. TW60_F: Ointment formulation containing 75 mM of the TBO that is incorporated with Tween60.

We tried to form various prototypes, to efficiently deliver the photosensitizer TBO into gingival sulcus region, with different molecular weight (g) ratio on photosensitizer: non-ionic detergent: plastibase for formulation condition. Span 20 and Tween 60 were assessed for suitability as non-ionic detergent and the release from Span 20 was more efficient than from the other (Fig. 4A–C). Finally the ratio was optimized at 1: 5: 100 for each component. The oral retention rate is an important feature of formulations designed for drug delivery to the oral cavity. The adhesive strength assessment showed that the retention properties of all the TBO formulations were not significantly different according to the prescriptions. In all formulations tested, the retention rate was higher than 93% at 180 min (Table 1).

**Table 1:** Drug retention test of TBO formulations.

TBO formulations	Percent drug retained on acrylic plate				
	0 min	30 min	60 min	120 min	180 min
TW60_D	100.0 (%)	98.1	94.6	95.8	93.0
SP80_F	100.0	103.1	101.6	100.5	101.1
SP20_F	100.0	98.4	98.2	98.9	99.0
TW60_F	100.0	99.4	98.7	99.9	100.8

The effect of the drug concentration on drug release was evaluated for each formulation containing 0.03 mM and 75 mM TBO. The F_formulation (75 mM) contained an approximately 2.3 times higher concentration of TBO than the D_formulation (0.03 mM) did (Table 2) The data showed that the TBO release from the formulation increased in a drug concentration-dependent manner. The TBO elution from the 0.03 mM formulation was below the limit of quantification at most time points, which suggests that the drug concentration affected the drug release.

**Table 2:** API concentrations and content of TBO formulations.

	TBO 0.03 mM^a^			TBO 75 mM^b^		
	Span80 (SP80-D)	Span20 (SP20-D)	Tween 60 (TW60-D)	Span80 (SP80-F)	Span20 (SP20-F)	Tween 60 (TW60-F)
**Materials**						
TBO (g)	0.12	0.12	0.13	0.28	0.28	0.28
Surfactant (g)	0.64	0.64	0.64	0.65	0.64	0.64
Plastibase (g)	10.01	10.12	10.15	10.16	9.98	10.24

^a^TBO 0.03 mM = 0.01 mg/mg = TBO 1%.

^b^TBO 75 mM = 22.94 mg/ml = 0.02294 mg/mg.

The release of TBO from formulations with the different surfactants, Span 80, Span 20, and Tween 60 was evaluated. Only the quantifiable results of formulations containing 75 mM TBO incorporated with Span 20 or Tween 60 (SP20_F and TW60_F, respectively) are presented here. The drug release over the experimental period increased in a time-dependent manner in the order of TW60_F and SP20_F (Fig. 4).

The normal saline elution medium was used to simulate standard of body fluids in the experiment described above; however, the elution rate was extremely low at 4.8 µg/mL (2.6%) at 3 h in the TW60_F formulation (Fig. 4A). To lower the polarity of the solvent, MeOH was added to the medium (100 mL PBS + 50 mL MeOH) and the TBO release was compared with and without MeOH. In the presence of MeOH, the TBO release was increased to up to 6.2 and 7.8 µg/mL (3.25 and 4.25%) in the SP20_F and TW60_F formulations, respectively at 3 h, when the formulations were applied to the acrylic plate (Fig. 4B). To reproduce actual oral cavity conditions during ointment application, the formulations were also applied to a porous sponge in addition to the acrylic plate, and the elution test was performed to measure the TBO release. Compared to the release profile of the formulations applied to the acrylic plate, a burst release of TBO was observed when the formulation was applied to the porous sponge. In the presence of MeOH, the TBO release from the TW60_F formulation that was applied on the sponge was increased up to 96.4 µg/mL (55%) in 3 h. The release from the SP20_F formulation on the acrylic plate was 34.1 µg/mL (19%) at 3 h (Fig. 4C).

In the viscosity analysis of TW60_F, the measured viscosity was 743666.7 mPa·s at 23.2 °C, and 603733 mPa·s at 30.3 °C (Table 3).

**Table 3:** The viscosity analysis of TW60_F.

	Viscosity (mPa·s)	Torque (%)	Speed (RMP)	Temperature (°C)
TW60_F	726000	60.5	1	23.3
	724600	61.9	1	23.2
	762400	63.5	1	23.1
	Warming ↓			
	570600	47.5	1	29.1
	641200	53.4	1	30.5
	599400	50.0	1	31.3

### *In vitro* antibacterial efficacy of TBO formulations (TW60_F)

**Figure 5:**
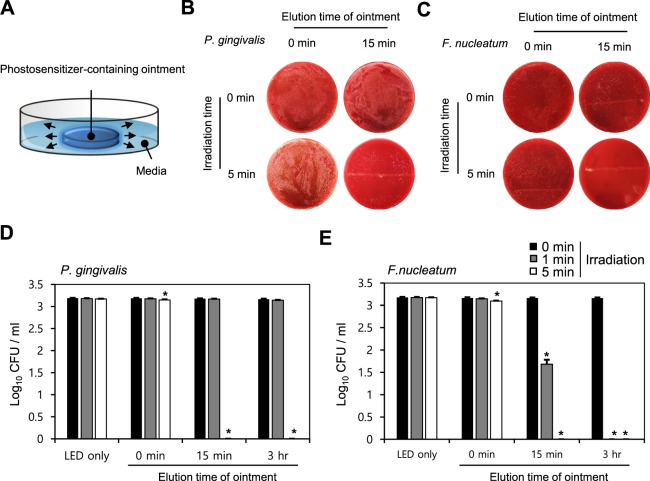
*In vitro* release profiles of TBO from various conditions and antibacterial test of TBO formulations (TW60-F) with LED irradiation. Release of TBO from SP20-F and TW60-F formulations. (**A**) TBO formulations were applied on an acrylic plate and eluted in saline medium. The experiments proceeded in the dark room. TW60-F was gently stirred with magnetic bar at 150 rpm for 0 min, 15 min and 3 h in culture media, then added to the culture wells to give final TBO concentrations of 0.33 mM. After 5 min incubation, (**B**,**D**) *P*. *gingivalis* and (**C**,**E**) *F*. *nucleatum* bacterial suspension wells were exposed to 60 mW/cm^2^ of LED at 650 nm wavelength for 1 min and 5 min. After irradiation, all the suspended bacteria was spread on an agar plate and incubated at 37 °C for 48–72 h. Then the viable bacterial colonies were counted. The data represent mean ± SD. (n = 2). *p < 0.05 vs. control (without TW60_F, without LED irradiation).

Based on the screening study results described above, the TW60_F formulation, which showed the highest elution rate was selected (Fig. 5A). The results of screening for the optimal combination, the combination of 0.33 mM TW60_F formulation with 60 mW/cm^2^ LED irradiation was evaluated for *in vitro* antibacterial activity. The mean CFU values of the colonies exposed to TW60_F-mediated LED photoactivation are analyzed. Incubation of *P*. *gingivalis* and *F*. *nucleatum* with the TW60_F in the dark showed no effect on bacterial growth. Irradiation of the bacterial suspension achieved a high photokilling rate (~99%) when *P*. *gingivalis* was irradiated with 60 mW/cm^2^ LED for 5 min in the presence of the TW60_F formulation (final TBO concentration: 0.33 mM) (Fig. 5B,D). Furthermore, irradiation of the *F*. *nucleatum* bacterial suspension at 60 mW/cm^2^ LED for 1 min in the presence of the formulation TW60_F (final TBO concentration: 0.33 mM) achieved a ~99.9% photo-killing rate (Fig. 5C,E).

### *In vivo* antibacterial efficacy of TBO formulations (TW60_F)

**Figure 6:**
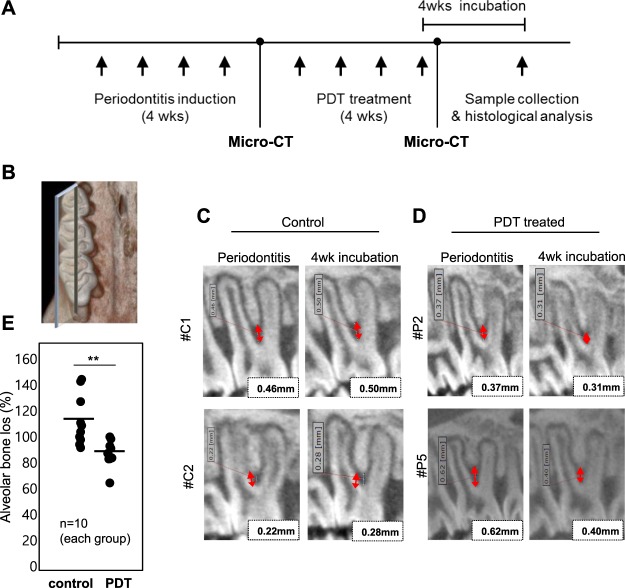
*In vivo* antibacterial test of TBO formulations (TW60_F) with LED irradiation. (**A**) Periodontitis induction schedule is depicted. Periodontitis induction was performed for 4 weeks with ligation suture and bacterial spreading. PDT was treated for another 4 weeks. (**B**) The animals were monitored by micro-CT and 3D image of left molars were reconstructed for analysis. Bone loss level of furcation area was measured at the time before and after treatment in (**C**) control and (**D**) PDT-treated group. (**E**) Percent change of bone loss was displayed.

Next, we tested whether this PDT, using TBO oral formulation with LED irradiation, is effective in an *in vivo* model. Periodontitis was induced in rats by silk ligation, and spreading of two species of bacteria, *P*. *gingivalis* and *F*. *nucleatum*, was repeatedly performed for 4 weeks (Fig. 6A). The combination of 0.33 mM TW60_F formulation with 60 mW/cm^2^ LED irradiation for 5 min was evaluated.

Mandibular alveolar bone loss at root furcation area was imaged by micro CT and analyzed using 3D viewer image analyzer (Fig. 6B). The alveolar bone loss observed in periodontitis induced control rats was attenuated in PDT treated rats (Fig. 6C,D). Increased distance from the furcation fornix to the intact interradicular bone (interradicular bone loss: red arrows) in periodontitis-induced control rats was significantly mitigated in the PDT-treated rats by 30% (p < 0.05) (Fig. 6E).

**Figure 7:**
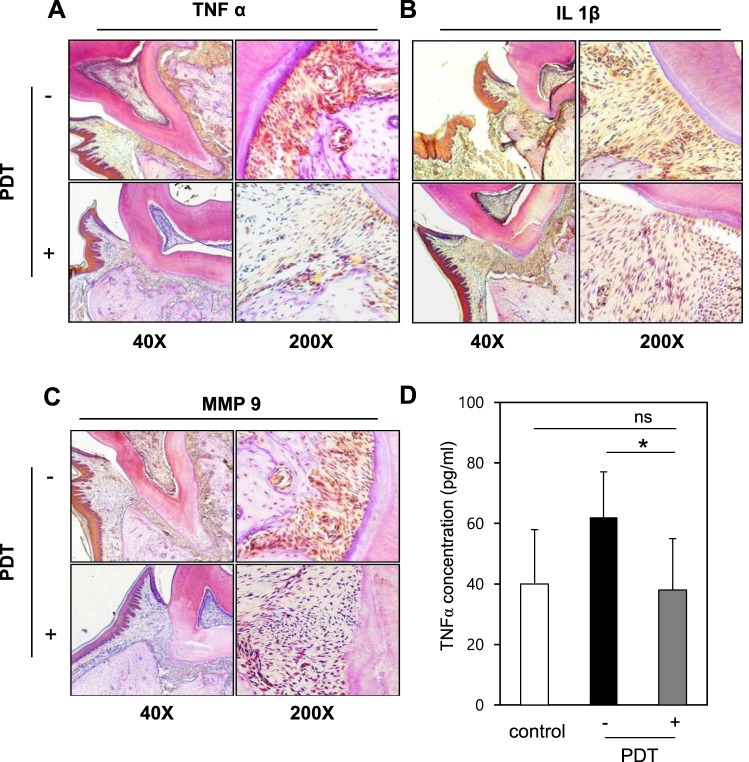
Analysis on inflammation and bone loss marker in animal samples. (**A**) TNF-α and (**B**) IL-1β and (**C**) bone absorption marker MMP-9 were analyzed by IHC. (**D**) The local concentration of inflammation was monitored with concentration of TNF-α in blood. *p < 0.05.

Because periodontitis is closely linked to the increased production of inflammatory cytokines, we examined the local expression and secretion of inflammation and bone destruction markers in periodontal tissue of rats. Immunohistochemical staining of TNF-α, IL-1β and MMP-9 in mandible sections yielded a much stronger staining intensity in gingiva and PDL area from periodontitis induced rats compared to control rats, but this was mitigated in PDT treated rats (Fig. 7A–C). An ELISA confirmed that the increased serum TNF-α level observed in periodontitis induced rats was significantly attenuated in PDT treated rats (Fig. 7D).

## Discussion

In the present study, we evaluated the efficacy of TBO-mediated PDT with LED irradiation to treat periodontitis. The penetrability of LED light at a wavelength of 650 nm through 3 mm-thick skin tissue and its activation of the antibacterial and anti-biofilm effect of TBO against *P*. *gingivalis* and *F*. *nucleatum* was demonstrated. Oral ointment formulation was optimized to enhance the delivery and oral targeting of TBO to the periodontal lesion; and the antibacterial efficacy of this TBO formulation mediated PDT was demonstrated in both *in vitro* and *in vivo* models.

TBO is a photosensitizer that can be activated by light at wavelengths between 600 and 650 nm, which means that TBO can be used as a suitable agent to absorb light at compatible wavelengths for the treatment of periodontitis. The depth penetration of light into a biological tissue is an important parameter for the correct determination of the irradiation dose in photodynamic therapy. Fodor *et al*. reported that light at 650 nm wavelength penetrates into the thickness of 3–4 mm human skin^21^. Bashkatov *et al*. investigated an optical properties of human skin, subcutaneous and mucous tissues in the wavelength range from 400 to 2000 nm^22^. For human mucous tissue, the absorption coefficient of light at 650 nm wavelength was close to zero and the scattering coefficient decreased as the wavelength increases. The optical penetration depth was estimated to be greater than about 3.5 mm at 650 nm. Given our result showing the transmission of light into deeper gingival target sites as well as previously proven advantages including limited heat production and reduced off target thermal effects, TBO-mediated PDT using LED irradiation seems to be a very effective combination as an adjunct therapy for persistent periodontitis.

Our *in vivo* study showed that the increased level of inflammation and bone destruction marker expression and secretion, which was significantly attenuated in the PDT-treated rats. These results confirm that LED with a 650 nm wavelength indeed penetrates the gingiva and reaches our TW60_F system (Tween 60 and plastibase-conjugated TBO topical formulation), which is sufficiently delivered to, and retained within, the gingival sulcus; thus, it correctly contacts the bacteria that reside around the gingival sulcus in periodontitis-induced rats.

Oral tissues are developed with blood vessels and have high mucosal permeability, which is favorable for topical application of drugs. However, their nature provides disadvantages, such as restriction of attachment to the necessary part for a long time by frequent movements of tongue and mucous membrane. The oral ointment formulation used in this study was based on the hydrophobic ointment base, plastibase, which is usually dissolved 5% of polyethylene resin in liquid paraffin under high temperature condition, and this formulation has little change in viscosity by temperature unlike conventional ointment bases. This property of the formulation is advantageous in maintaining the formulation applied over a period of time in the oral cavity, however, it is an adverse environment in which the aqueous toluidine blue O elutes from the formulation. Thus, we tried to improve the disadvantages of this ointment base using the surfactants, Span 20, Span 80 and Tween 60, having different HLB values on the elution of toluidine blue O from the same ointment base. The TBO formulations synthesized by conjugation with plastibase and one of the three types of surfactants (Span 20, Span 60, or Tween 60) were evaluated for their retention ability, drug release rate, and antimicrobial activity. Screening for the suitable surfactant and the optimization study revealed that the TW60_F formulation was the optimal composition for superior drug release over a short period. In the viscosity analysis of TW60_F, the measured viscosity was 743666.7 mPa·s at 23.2 °C, and 603733 mPa·s at 30.3 °C, which is over five to seven times higher than that of common oral ointments (Table 3). The *in vitro* antibacterial test results demonstrated that the TW60_F formulation containing TBO effectively reduced the viability of *P*. *gingivalis* and *F*. *nucleatum*. The major difficulty in developing a topical formulation for the eradication of oral bacteria is the dilution and or rapid elimination of the drug by the flushing action of saliva^23^. In the present study, all the TBO formulations tested showed a ≥93% retention rate at 180 min. The adhesive strength of these formulations would likely allow the delivery of TBO to target sites, including the gingival sulcus, under conditions affecting the drug residence time, including the shear force associated with speaking, swallowing, mastication, and the washout induced by saliva production.

The results of present study indicate that drug release is dependent on a number of manipulatable variables. The release amount is dependent on the amount of the drug incorporated into the formulation, surfactant, and external conditions including physical irritation or solvent polarity. By manipulating these factors to optimize them, the drug delivery can be customized to enhance the release of TBO over a short period. The drug elution from the porous sponge was remarkably higher than that from the acrylic plate. The presence of MeOH in the elution medium increased the TBO release from the TW60_F formulation to up to 55% in 3 h. This data implies that drug release could be increased by adequately exposing the formulation to saliva or physical irritation in the actual oral environment. The *in vitro* antibacterial test results, which showed that the drug was sufficiently eluted to induce a 99.9% bactericidal rate within 15 min, also supported this supposition.

The exposure of bacterial cultures to LED light in the presence of TBO led to a dose-dependent reduction in *P*. *gingivalis* and *F*. *nucleatum* viability. In the context of light intensity, there was a significant decrease in colony counts when the light intensity was increased from 3 mW/cm^2^ to 60 mW/cm^2^. When the exposure to LED irradiation was increased from 1 min to 5 min, the bactericidal effect increased for all tested microorganisms. In the context of TBO concentration, higher concentrations of TBO (0.98 mM) resulted in a significant reduction in the viability of the bacterial culture after 48 h of incubation without light exposure, demonstrating direct toxicity with TBO as a sensitizer.

Taken together, our findings indicate that TBO-mediated PDT with LED irradiation has the potential to serve as a safe adjunctive antimicrobial procedure for nonsurgical periodontal treatment. The Tween 60 and plastibase-conjugated TBO topical formulation can be used as prototype for new antimicrobial PDT development and clinical application for periodontitis treatment.

## Materials and Methods

### Bacterial strains and culture conditions

*Porphyromonas gingivalis (P*. *gingivalis*; ATCC 33277) and *Fusobacterium nucleatum (F*. *nucleatum*; ATCC 25586), which are common causative agents in periodontitis, were obtained from the Korean Collection for Type Culture (KCTC, Daejeon, Korea). *P*. *gingivalis* and *F*. *nucleatum* were incubated on an agar plate enriched with tryptic soy broth (Becton, Dickinson, and Company, MD, USA) containing 5% sheep’s blood (Komed, Seongnam-si, Korea), 5 µg/mL hemin (Sigma Aldrich, MO, USA), and 0.1 µg/mL menadione (vitamin K1) (Sigma Aldrich, MO, USA) for 48–72 h at 37 °C in an anaerobic workstation. Next, a bacterial colony was re-suspended in a 5 mL suspension solution at 37 °C for 48–72 h. The number of cells was adjusted by means of a spectrophotometer (Ultrospec 3000pro, Amersham-Pharmacia, Uppsala, Sweden) at a wavelength of 540 nm (*P*. *gingivalis*: 1 × 10^10^ cells/mL; *F*. *nucleatum*: 1 × 10^6^ cells/mL). A 100 µL sample of adjusted bacterial suspension was placed into the wells of a 96-well strip immunoplate (SPL Life Sciences, Gyeonggi-do, Korea) for photodynamic activation.

### Photodynamic activation studies

All experiments were performed in a dark room. TBO in powder form (Sigma Aldrich, MO, USA) was dissolved in phosphate-based saline (PBS). Five µL of various concentrations of TBO solutions were added to the wells to obtain final concentrations of 0.16, 0.33, and 0.98 mM. After 5 min of incubation, TBO-containing wells (total volume 100 μl) were exposed to the LED light. A light-emitting diode device (MK3003D, MKPOWER, Seoul, Korea: power output of 90 mW) with a wavelength of 650 nm was used. LED light delivered using a custom-designed adapter and a fiber-optic cable. The power density passing through wells were 3, 30, and 60 mW/cm^2^, respectively. The duration of irradiation was 1 min and 5 min. TBO-negative controls were incubated with an equal volume of phosphate buffered saline (PBS). The distance between the LED and the plate was 1 cm. After LED irradiation, all the suspended bacteria were spread on an agar plate and incubated at 37 °C for 48–72 h in accordance with the incubation conditions. Then, the bacterial colonies were counted and converted into Log_10_ CFU/ml.

To test the whether 650 nm wavelength LED light penetrates through tissues that are similar in thickness to gingival tissues and activates the bactericidal effect of TBO, 3-mm-thick artificial skin (Geistlich Mucograft®, Geistlich Pharma, NJ, USA) was placed between the LED device and the bacterial suspension well. LED penetration experiments were performed in the same manner as described above.

The antibacterial activity of TBO formulations was evaluated as follows. The formulation containing 75 mM TBO incorporated with Tween 60 (TW60_F) was gently stirred using a magnetic stirring bar at 150 rpm for 0 min, 15 min, and 3 h in the culture media, and then the mixture was added to the culture wells to obtain final TBO concentration of 0.33 mM. After a 5 min incubation, the wells were exposed to 60 mW/cm^2^ LED at a 650 nm wavelength for 1 and 5 min. Then, the negative controls for TW60_F were incubated with equal volume of PBS. The distance between the LED and the plate was 1 cm, and all these experiments were performed in a dark room. After LED irradiation, all of bacterial cells in 96-well plate was spread on a tryptic soy agar plate and incubated at 37 °C for 48–72 h in accordance with the incubation conditions. After incubation, the bacterial colonies were counted and converted into Log_10_ CFU/ml.

### Determination of biofilm disruption

Bacterial biofilm was formed on 96-well microtiter plates (SPL Life Sciences, Gyeonggi-do, Korea). *P*. *gingivalis* and *F*. *nucleatum* were incubated in saliva-coated 96-well strip immunoplates at 37 °C for 48–72 h in an anaerobic workstation. For 96-well strip immunoplate coating, saliva was collected from healthy humans and mixed with 1 × PBS (1:9 v/v) to perform centrifugation for 10 min at 3000 rpm at 4 °C. The study protocol and experimental design for collection of saliva from healthy humans was approved by the Wonkwang University Dental hospital IRB, approval ID: WKDIRB201708-01. The supernatant was filtered using 0.22 μm syringe; the 96-well strip immunoplates were coated at 4 °C for 4 h, and then stored at 4 °C until use. The established biofilm was treated with PDT through 3 mm-thick artificial skin in accordance with the same protocol and incubated for 24 h using the same incubation condition described above in the photodynamic activation studies section.

For crystal violet staining, the culture supernatant was discarded and the wells were washed twice with 1 × phosphate-buffered saline (1 × PBS). The remaining biofilm cells attached to the polystyrene surface were fixed with 95% ethanol for 10 min and stained with 0.1% crystal violet for 15 min. Plates were then washed twice with 1 × PBS and air-dried. For quantitative measurement of the biofilm, the bound crystal violet was solubilized with 10% glacial acetic acid for 10 min and OD595 detected using Microplate Spectrophotometer (BioTek, St, Winooski, VT, USA).

### Preparation of TBO topical formulations

According to the categorization by Katz and Ariens (M. Katz, E.J. Ariens (Ed.), Drug design, Academic Press, New York, 1973), ‘Absorption base’ releasing drug above certain time is suitable formulation for oral cavity. This formulation is consist of oil phase, such as hydrocarbon type organogel or hydrophilic organogel, and water in oil type detergents, and should be optimized depending on characteristics and purpose of drug. The formulations were composed of three main ingredients, toluidine blue O (Sigma-Aldrich, MO, USA), surfactant (Span 80, Span 20, or Tween 60), and ointment base (plastibase). To test the various hydrophilic-lipophilic balance (HLB) value prescriptions, the surfactants selected were Span 80, Span 20, and Tween 60 (Samchun Pure Chemical, Gyeonggi, Republic of Korea). TBO formulations containing different concentrations of TBO (0.03 mM or 75 mM) and ingredients were prepared as follows. The weighed amounts of TBO were slowly added to the surfactant with gentle mixing. After a clear viscous solution had been formed, the TBO solution was mixed into the plastibase by applying geometric dilution method. Once dissolved, the solubility decreased as TBO solution is mixed with the base, and the drug particles were formed in a ‘bottom-up’ manner. The size of the particles formed was about a few tens μm in size, in a homogeneous state. TBO reacts with light, therefore, it was prepared under shaded conditions. The various TBO formulations developed with different compositions were coded as follows: 0.03 mM: SP80_D, SP20_D, and TW60_D; and TBO 75 mM: SP80_F, SP20_F, and TW60_F; Table 2. The composition of the formulations was determined based on the publication “Drug Design”^24^.

### *In vitro* release from sponge or acrylic plate

To evaluate TBO release from formulations, elution assays were conducted after uniform application of 1.2 g of each formulation to 2.0 cm × 2.0 cm area, 1 cm away from both ends of 2 cm × 4 cm size sponge, or 0.8 g of each formulation to 2.0 cm × 2.0 cm area, 1 cm from both ends of 2 cm × 12 cm size acrylic plate. We placed the sponge or acrylic plate into 150 mL elution medium consisting of saline or mixed medium (100 mL of phosphate-buffered saline (PBS) with 50 mL methyl alcohol [MeOH]) at 36.5 °C. The sponge was allowed to float so that the solvent could reach the ointment surface, and the acrylic plate was set obliquely on a beaker. Samples were gently agitated using a magnetic spinning bar at 200 rpm for sponge and 150 rpm for acrylic plate. Samples were withdrawn at intervals of 0, 1, 3, 8, 15, 30, 60, 120, and 180 min. and spectrophotometrically analyzed TBO content using an ultraviolet (UV) - visible detector.

### *In vitro* drug retention and viscosity test

To test the retention rate of a drug in the oral cavity, retention test was performed. After conducting the experiment described above, acrylic plate samples were collected at 0, 30, 60, 120 and 180 min. The retention rate was obtained by weighing the acrylic plate after removing the water of the acrylic plate. Viscosity was measured with a Brookfield DV-II + Viscosimeter (Brookfield, USA). Formulation was heated from 23.3 °C ± 0.1 °C to 30.45 ± 0.96 °C in a measuring cylinder. The same spindle (spindle LV-5) and the same stirring rate (1 rpm) were used for each experiment.

### Animals and experimental periodontitis model

The study protocol and experimental design were approved by the Animal Ethics Committee of Wonkwang University, approval ID: WKU17-64. Thirty of 6 weeks old male Wistar rats were used in the experiment. Their body weights ranged from 300 to 330 g at the beginning of the experiment. The animals were randomly divided into three groups (n = 10): non-ligated; ligature only; ligature plus PDT. Periodontitis induction was performed once a week for 4 weeks by ligating suture and spreading bacteria. A 4-0 silk suture was placed submarginally around the first molar in both mandibular quadrants. The animals received oral inoculations of a fresh 0.1 ml broth culture of a strain of *P*. *gingivals* and *F*. *nucleatum* on targeted gingival region. The sutures were checked after application and lost or loose sutures were replaced. PDT was conducted once a week for 4 weeks. TBO ointment was applied to the gingival sulcus of the periodontal lesion and left to be permeated for 10 min. The gingiva was exposed to LED light with the same condition as *in vitro* (10 mW/cm^2^, 650 nm) for a 5 min. After another 4 weeks after the last PDT treatment, the animals were sacrificed, and the mandible was dissected free of the muscles and the soft tissue, keeping the attached gingiva intact with the bone. At the same time, animal blood samples were collected. Micro-CT images of the gingival region were scanned four times: one before the induction of periodontitis and the other three at the end of each phase (inducing phase, PDT phase, incubation phase). Including periodontitis induction and PDT, all proceses with the animals were performed under general anesthesia using ketamine (40 mg/kg) through intraperitoneal injection. All experiments were performed in accordance with relevant guidelines and regulations.

### Micro-CT imaging

CT imaging was performed using a Quantum GX μCT imaging system (PerkinElmer, Hopkinton, MA, USA), located at Korea Basic Science Institute (Gwangju, Korea). The X-ray source was set to levels of 90 kV and 88 µA with a field of view of 45 mm (voxel size, 90 μm; scanning time, 4 min). The CT imaging was represented by 3D Viewer, existing software within the Quantum GX. All images were typically exported individual slices in a file DICOM format for medical imaging.

### Immunohistochemistry

Isolated tissue sections were decalcificated and then paraffinized. Deparaffinized tissues were blocked with 3% H_2_O_2_ phosphate-buffered saline to inactivate endogenous peroxidases. Slides were washed with phosphate-buffered saline, incubated for 20 min in protein-blocking solution supplemented with 4% normal bovine serum albumin, incubated overnight at 4 °C with primary antibodies against TNF-α (Cat. No. sc-1351), IL-1β (Cat. No. sc-1252) and MMP-9 (Cat. No. sc-6841), which were purchased from Santacruz Biotechnology (California, US). Anti-Mouse polymer kit (Dako, Japan) was used for DAB development. The tissues were counterstained with hamatoxylin.

### Statistical analysis

The results were expressed as mean values ± standard deviations (mean ± SD). A two-way analysis of variance (ANOVA) was performed with post hoc testing (Tukeys’ test) as appropriate to determine whether there were significant differences among the test conditions. To analyze serum TNF-α variables, one-way ANOVA was performed with post-hoc testing as appropriate. A *p*-value < 0.05 was considered statistically significant.

## Supplementary information

Supplementary Figure 1
