# Supplementary material for: Non-Invasive Photodynamic Therapy against -Periodontitis-causing Bacteria
Source: Sci Rep. 2019 Jun 3;9:8248. doi: 10.1038/s41598-019-44498-4 (PMC6546788; doi:10.1038/s41598-019-44498-4)
Supplement: Supplementary file 1 — Supplementary Figure 1 [file 41598_2019_44498_MOESM1_ESM.pdf]

Non-Invasive Photodynamic Therapy against -Periodontitis-causing Bacteria

Danbi Park [a,†], Eun Joo Choi [b,†], Kwon-Yeon Weon [c,†], Wan Lee [b], Seoung Hoon Lee [b], Joon-Seok Choi [c], Gyu Hwan Park [d], Bada Lee [e,f], Mi Ran Byun [e], Kyunghwa Baek [a,*] and Jin Woo Choi [e,f,*]

[a] Department of Pharmacology, College of Dentistry and Research Institute of Oral Science, Gangneung-Wonju National University, Gangwon-do, 25457 Republic of Korea;

[b] School of Dentistry and Dental Research Institute, Wonkwang University, Iksan, Choenbuk, 54538, Republic of Korea;

[c] College of Pharmacy, Daegu Catholic University, Gyeongbuk, 38430, Korea;

[d] Research Institute of Pharmaceutical Sciences, College of Pharmacy, Kyungpook National University, Daegu 41566, Republic of Korea;

[e] Department of Pharmacology, College of Pharmacy, Kyung Hee University, Seoul 02453, Republic of Korea;

[f] Department of Life and Nanopharmaceutical Sciences, Kyung Hee University, Seoul 02453, Republic of Korea

Fig. S1

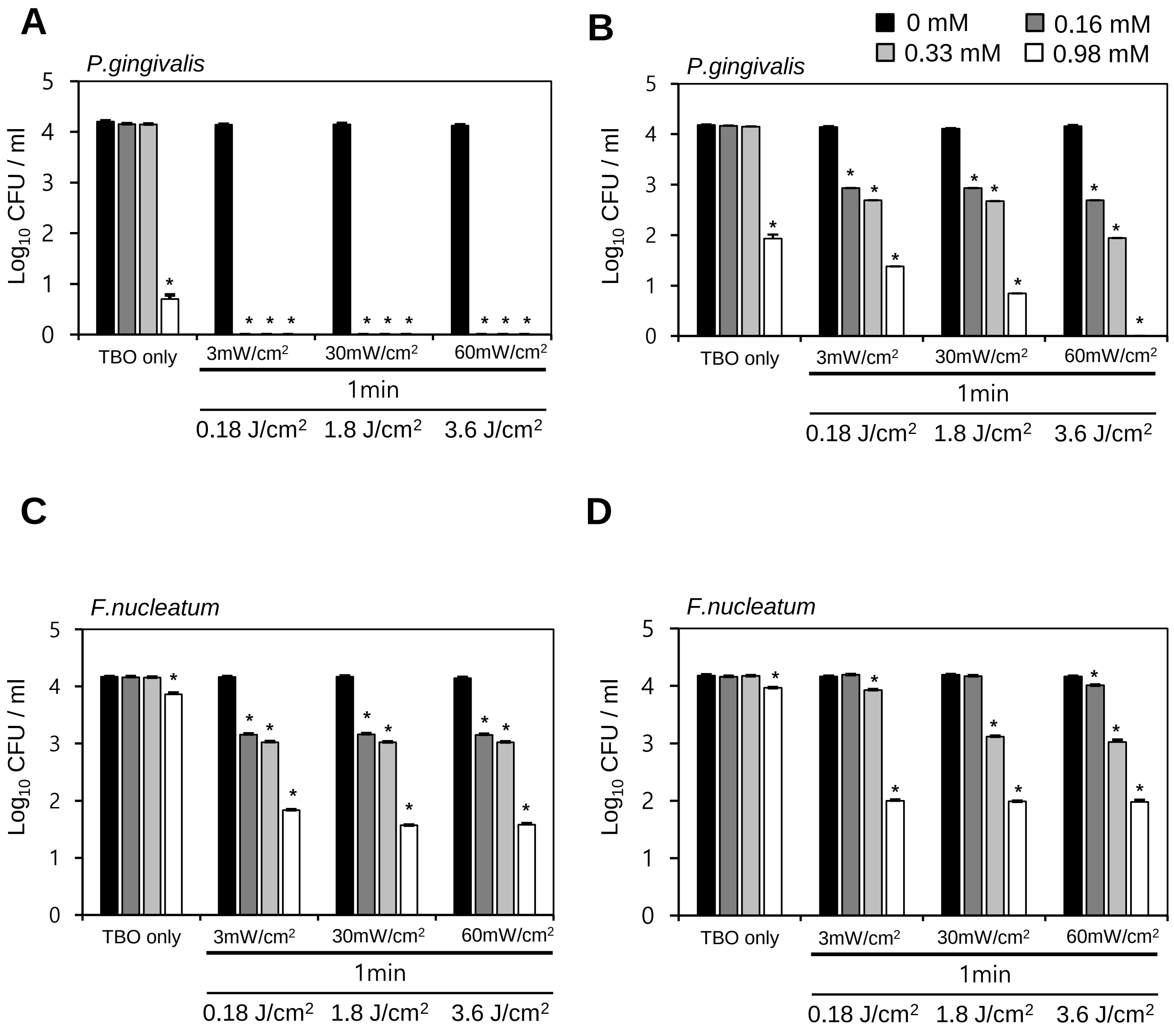

Fig. S1 Tissue-penetrating and TBO-activating effect of 650 nm LED

TBO in powder form was dissolved in PBS. TBO solutions were added to the bacterial suspension to obtain final concentrations of 0.16, 0.33, and 0.98 mM. After 5 min of incubation, TBO - containing wells were exposed to 3, 30, and 60 mW/cm^2 of LED irradiation at a 650 nm wavelength for 1 min. TBO - containing bacterial suspension wells were directly exposed to LED irradiation (A) for *P. gingivalis* and (C) for *F. nucleatum*. TBO - containing bacterial suspension wells were exposed to LED irradiation through 3 mm-thick artificial skin (B) for *P. gingivalis* and (D) for *F. nucleatum.* The data represent the mean ± the standard deviation (n = 2), * $p < 0.05$, ** $p < 0.01$ vs. control (without TBO and without LED exposure). CFU, viable count colony forming unit
